# Commentary: Research on the allocation of 3D printing emergency supplies in public health emergencies

**DOI:** 10.3389/fpubh.2025.1670116

**Published:** 2026-01-13

**Authors:** Sajitha R. Nair, Praveen Nagarajan, Santosh G. Thampi, Rajesh P.

**Affiliations:** 1 NSS College of Engineering, Palakkad, India; 2 National Institute of Technology, Calicut, India

**Keywords:** 3D printing, additive manufacturing, emergency logistics, public health emergencies, NSGA-II algorithm, sustainable supply chains, emergency supplies allocation

## 1 Introduction

The COVID-19 pandemic exposed major weaknesses in conventional medical supply chains, particularly their limited ability to respond to sudden surges in demand for personal protective equipment and critical medical items (1). In this context, three-dimensional printing (3DP) emerged as a flexible, decentralized, and rapid manufacturing alternative capable of addressing urgent supply shortages. The study by He et al. (2) represents a significant contribution to this field by developing a multi-objective optimization model that matches 3DP equipment with emergency production tasks using an improved NSGA-II algorithm (3). This commentary evaluates the methodological strength, practical relevance, and innovation of the authors' approach while also identifying key areas in which future work can expand, particularly regarding sustainability, dynamic demand, and integration with smart-logistics systems. The aim is to highlight both the theoretical value and real-world implementation opportunities of the model and to situate it within emerging conversations on resilient and low-carbon emergency manufacturing.

### 1.1 Evaluation of core contributions

A major strength of the study lies in its realistic representation of emergency production environments. The model integrates diverse operational constraints—including device capacity, material compatibility, order requirements, processing speed, transportation cost, and spatial configuration—into a unified optimization framework. The enhanced NSGA-II algorithm, featuring a normal distribution-based crossover operator and dynamic crowding distance mechanism (4), effectively addresses common challenges in evolutionary algorithms, such as premature convergence and reduced Pareto diversity (9). The demonstrated improvement over traditional NSGA-II confirms the robustness of the proposed enhancements. The authors also provide detailed differentiation among 3DP technologies—SLA, SLS, FDM, and 3DP—allowing for a more accurate match between production tasks and equipment capabilities (5). This granularity is particularly valuable in emergency contexts where precision, speed, and material compatibility directly affect usability of the produced items. However, environmental considerations could be strengthened. Additive manufacturing is frequently framed as a sustainable alternative due to reduced material waste (10), yet energy consumption, emissions, and end-of-life recovery vary widely across materials and technologies. Incorporating such data could support a more comprehensive assessment of the environmental impact of emergency 3DP deployment (6).

## 2 Discussion

The contributions outlined above extend naturally toward broader themes of emergency preparedness, resilience planning, and sustainable logistics systems (7). He et al.'s model demonstrates strong potential for operational use in decentralized emergency manufacturing networks—especially in situations where regional production capacities differ and rapid response is essential. Their algorithm provides a technically rigorous foundation for matching supply and demand during crises. Nonetheless, opportunities exist to further enhance the model's practical utility. Integrating GIS-based spatial data layers could enable dynamic rerouting and real-time adaptation based on road closures, weather disruptions, or shifting population needs. For example, adding geographic congestion weights to the transportation cost function could better reflect spatial accessibility constraints during emergencies. Similarly, incorporating low-carbon and recyclable materials into the allocation model could align emergency manufacturing with global sustainability goals. Assigning carbon-intensity scores or material-recyclability weights to decision variables would allow the model to generate environmentally optimized Pareto fronts. Further, 3DP units deployed during emergencies could later serve dual purposes—producing modular construction components for temporary clinics, shelters, or community infrastructure (8). Including such dual-use scenarios could enhance long-term system resilience and justify investment in mobile or distributed 3DP networks.

## 3 Conclusion

The work by He et al. provides a timely and technically insightful framework for matching 3D printing equipment to emergency supply needs during public health crises. By integrating realistic operational parameters with an enhanced multi-objective optimization algorithm, the study offers a valuable contribution to emergency logistics and additive manufacturing research. Moving forward, incorporating environmental sustainability metrics, life-cycle perspectives, and adaptive geospatial data would further expand the model's relevance to modern low-carbon and smart-resilient logistics systems. Attention to dynamic demand, recyclable materials, and dual-use deployment scenarios would also enable more comprehensive planning for future emergencies. Overall, this commentary underscores the importance of bridging advanced optimization techniques with sustainable, flexible, and socially responsive manufacturing systems—an essential step toward strengthening global resilience in the face of increasingly complex public health challenges.
